# Graphene Oxide Membranes for Trace Hydrocarbon Contaminant Removal from Aqueous Solution

**DOI:** 10.3390/nano10112242

**Published:** 2020-11-12

**Authors:** Alessandro Pedico, Marco Fontana, Stefano Bianco, Seifeddine Kara, Monica Periolatto, Stefano Carminati, Candido Fabrizio Pirri, Elena Tresso, Andrea Lamberti

**Affiliations:** 1 Politecnico di Torino, Dipartimento di Scienza Applicata e Tecnologia (DISAT), Corso Duca degli Abruzzi, 24, 10129 Torino, Italy; marco.fontana@polito.it (M.F.); stefano.bianco@polito.it (S.B.); seifeddine.kara@polito.it (S.K.); monica.periolatto@polito.it (M.P.); fabrizio.pirri@polito.it (C.F.P.); elena.tresso@polito.it (E.T.); andrea.lamberti@polito.it (A.L.); 2 Istituto Italiano di Tecnologia, Center for Sustainable Future Technologies, Corso Trento, 21, 10129 Torino, Italy; 3 Eni S.p.A., Piazza Ezio Vanoni, 1, 20097 San Donato Milanese, Italy; Stefano.Carminati@eni.com

**Keywords:** graphene oxide, membranes, oil and gas, BTX, rejection, trace contaminants, water purification, filtration, solvents, wastewater

## Abstract

The aim of this paper is to shed light on the application of graphene oxide (GO) membranes for the selective removal of benzene, toluene, and xylene (BTX) from wastewater. These molecules are present in traces in the water produced from oil and gas plants and are treated now with complex filtration systems. GO membranes are obtained by a simple, fast, and scalable method. The focus of this work is to prove the possibility of employing GO membranes for the filtration of organic contaminants present in traces in oil and gas wastewater, which has never been reported. The stability of GO membranes is analyzed in water solutions with different pH and salinity. Details of the membrane preparation are provided, resulting in a crucial step to achieve a good filtration performance. Material characterization techniques such as electron microscopy, x-ray diffraction, and infrared spectroscopy are employed to study the physical and chemical structure of GO membranes, while gas chromatography, UV-visible spectroscopy, and gravimetric techniques allow the quantification of their filtration performance. An impressive rejection of about 90% was achieved for 1 ppm of toluene and other pollutants in water, demonstrating the excellent performance of GO membranes in the oil and gas field.

## 1. Introduction

**Figure 1:**
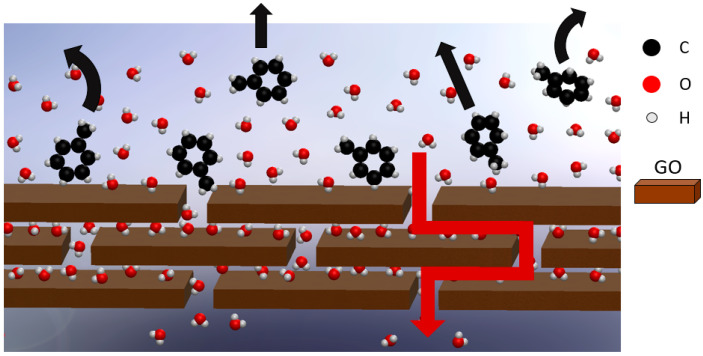
Working principle of graphene oxide (GO) membranes. Here is a sketch of the stacked GO structure, in which water molecules are free to cross the membrane (red arrow) while toluene molecules are rejected (black arrows).

Graphene-based materials, including single-layer graphene and derivatives such as graphene oxide (GO), have received growing attention from the scientific community in the past few years [1]. Many studies can be found in the literature about graphene-based membranes applied for water purification, such as single-layer graphene with controlled pores. This technology, initially theorized [2] and then experimentally realized [3] for water desalination, is so far under development because of the great effort required to achieve controlled filtration properties together with scalable and low-cost preparation techniques [4]. These problems move the spotlight to GO and its reduced form with a lower oxygen content (rGO) [5]. Based on the findings from numerical simulations [6,7,8], many experimental studies have been reported in the literature, showing the possibility of tuning GO’s properties to achieve good filtration results [9,10,11]. In this context, GO membranes find application for their natural hydrophilicity and intrinsic stacked structure, constituting two-dimensional channels whose height is slightly less than 1 nm. Such membranes, indeed, are made of a stack of flakes packed up to form a layered structure which is responsible for the filtration properties, as schematically reported in Figure 1.

These particular features make them promising as nanofiltration (NF) membranes for ultra-selective separation [12,13,14,15,16,17] and water desalination [18,19,20,21], and also in tubular supports [22,23]. GO membranes are also renowned for their antifouling properties [24,25]. Furthermore, a low-cost, safe, and scalable preparation method has recently been proposed [26]. Innovative membranes based on graphene and other 2D materials [27,28] also offer interesting possibilities for a variety of applications in the oil and gas industry [29], ranging from sulfate removal and the tailoring of the water chemistry for improved oil recovery (IOR) and enhanced oil recovery (EOR) to the desalination/purification of produced water or industrial wastewater for recycling and discharge to the environment.

Among all the hydrocarbon contaminants in produced water, benzene, toluene, and xylene (BTX) represent a key challenge for the oil and gas industries [30], which have to more efficiently purify their wastewater to meet the ever more stringent targets set by governments all over the world (see Table S1 in the Supporting Information for reference values for Italy and the USA).

To the best of our knowledge, all other works found in the literature (reported in Table 1) which focused their attention on hydrocarbons with a low solubility in water limit their analysis to solutions in which the pollutant is not dissolved in water but simply dispersed, such as emulsions with stabilized droplets [31,32,33,34]. Such experimental conditions strongly reduce the impact of the findings on the oil and gas field, since the same goal can be achieved using polymeric [35,36] and ceramic [37,38] ultrafiltration membranes or other commercial solutions already employed by oil and gas companies [39]. Distillation can be employed to remove many contaminants, but BTX has a boiling point close to water’s one. Activated carbon columns are employed to absorb most of the residual contamination present in stream, but they are not effective for pollutants whose concentration is far below the solubility limit. Moreover, such columns highly hinder water flux. If reverse osmosis can always be employed as a final step to obtain pure water, this is not the most cost-effective solution due to the required high pressure and membrane cost.

The aim of the present study is to give insight into the filtration properties of GO membranes, focusing attention on the possibility of selectively removing traces of BTX. For this purpose, we selected toluene as representative of the BTX class, since it is not carcinogenic like benzene and does not have isomers like xylene, making both the experimental section and the detection step simpler without affecting the potentiality of the results. In particular, the focus of this work is on using an NF GO membrane to go beyond the purification of oil-in-water emulsions previously discussed, filling a gap which is actually present in the literature. We experimentally verified the performance of GO membranes in the filtration of solutions containing organic pollutants in concentrations well below their solubility limit in water. Therefore, herein is reported a comparison of the selectivity towards many liquid pollutants present in the oil and gas produced water, together with details on the optimization of GO membrane fabrication in order to achieve an efficient rejection.

## 2. Materials and Methods

### 2.1. Starting Materials

Graphene oxide flakes (single-layer GO, 300~800 nm lateral dimensions, Cheap Tubes Inc., Grafton, VT, USA) are dispersed in deionized water (Direct-Q 3 UV, Merck Millipore, Burlington, MA, USA), testing different concentrations, in a range 0.1~5.0 mg/mL. Each solution is sonicated for 30 min using a frequency of 40 kHz in an ultrasonic bath (LBS2, FALC INSTRUMENTS SRL, Treviglio, Italy).

Different polymeric supports were tested: cellulose nitrate (Sartorius Stedim Italy S.r.l., Grassina, Italy), hydrophilic polycarbonate track etched (PCTE, Sterlitech, Kent, WA, USA), polypropylene (PP, Sterlitech, Kent, WA, USA), anodized alumina oxide (AAO, Sterlitech, Kent, WA, USA), and polyether ether ketone (PEEK, Sterlitech, Kent, WA, USA) with nominal pore dimension of 450, 100, 100, 20, and 5 nm, respectively. The diameter is equal to 47 mm for all the supports to match the size of the filtering apparatus.

The chemical reagents employed for the tests and characterizations of the GO membranes are toluene (anhydrous, 99.8% purity), methylcyclohexane (MCH, anhydrous, 99.0% purity), ethylene glycol (EG, anhydrous, 99.8% purity), triethylene glycol (TEG, anhydrous, 99.0% purity), methanol (anhydrous, 99.8% purity), ethanol (anhydrous, 99.8% purity), acetone (anhydrous, 99.5% purity), sodium chloride (anhydrous, 99.0% purity), sodium hydroxide (anhydrous, 97.0% purity), hydrochloric acid (36.5~38%), nitric acid (70%), Oil Red O (75%), and methylene blue (MB, 82%). All of them are supplied by Sigma-Aldrich, St. Louis, MO, USA.

### 2.2. Methodology

A gas chromatography flame ionization detection (GC-FID) method combining solid-phase microextraction (SPME) and static extraction (SE) was developed for the direct quantitative analysis of toluene, MCH, and glycols in water. SPME is a technique relying on the ability of a solid sorbent (commonly a thin fiber) to catch specific target molecules from a liquid phase. Once the analytes are captured, they can be released in gaseous form through thermal evaporation in a small closed volume. Once equilibrium is reached, the gas is sent to the column for the analysis. These are the principle of the SE method. For toluene and MCH, SPME was fast and efficient; SE is performed using a 100 µm polydimethylsiloxane fiber (Supelco, Belmont, PA, USA) with a 15 min static extraction time and a 2 min thermal desorption time. An Agilent HP 5890 gas chromatograph equipped with a flame ionization detector was used for separation and analysis. Separation was carried out using a 60 m × 0.32 mm i.d. capillary column (Supelco). The chromatographic conditions are as follows: detector 250 °C, injector 200 °C, column 150 °C for glycol or 120 °C for toluene and MCH. The flow rates adopted for each gas are: He carrier plus makeup 30 mL/min; air 300 mL/min; H**_2_** 3.0 mL/min. Gravimetric techniques, evaluating the density and the mass conservation by means of a micro-balance, are employed for the other compounds used at higher concentrations.

### 2.3. Lab Setup of Membrane Module

GO membranes were produced in two different apparatus—one working in vacuum conditions, with the other working with an applied overpressure. The apparatus for the vacuum filtration was supplied by VWR, while the apparatus for the pressure-driven filtration, exploiting the dead-end configuration, was the model HP4750, supplied by Sterlitech (see Figure S1 in the Supporting Information). The filtration experiments were performed in the dead-end apparatus. The pressure applied for the filtration tests was 1 bar, even if a pressure up to 25 bar was tested to verify the GO membrane strength. A nitrogen tank was employed as pressure source. A schematic representation of the setup is reported in Figure S2 in the Supporting Information.

### 2.4. Characterization

X-ray diffraction (XRD) spectroscopy (X’Pert pro, Malvern Panalytical, Malvern, UK) was employed to measure the interlayer distance between GO sheets in the stacked membrane. Exploiting the well-known Bragg’s law, the instrument was set to work with a Bragg–Brentano configuration, exploiting a Cu-Kα source with λ = 1.541874 Å. The measurements were performed with a step size of 0.026 degrees at a scan speed of 200 s/step.

Electron microscopy characterization was carried out with a field-emission scanning electron microscope (FESEM Supra 40, manufactured by Zeiss, Oberkochen, Germany) equipped with a Si(Li) detector (Oxford Instruments, Abington, UK) for energy-dispersive X-ray spectroscopy.

Fourier transform infrared (FTIR) spectroscopy (Nicolet 5700 FTIR, Thermo Fisher Scientific, Waltham, MA, USA) was performed directly on the GO membranes in an attenuated total reflection (ATR) configuration, using a step size of 0.4 cm^−1^ and a scan speed of 6.33 cm/s. The Z-potential was measured by Zetasizer Nano ZS90 (Malvern Panalytical, Malvern, UK) for solutions of GO with a concentration of 0.05 mg/mL. Both techniques were employed to have a confirmation of the presence of negatively charged groups on the GO surface [42].

UV-visible spectroscopy (Lambda 35, PerkinElmer, Waltham, MA, USA) has been employed to evaluate the remaining fraction of MB in solution. The measurements were run with a selected spectral bandwidth of 0.5 nm at a speed of 30 nm/min.

## 3. Results and Discussion

### 3.1. Membrane Fabrication

**Figure 2:**
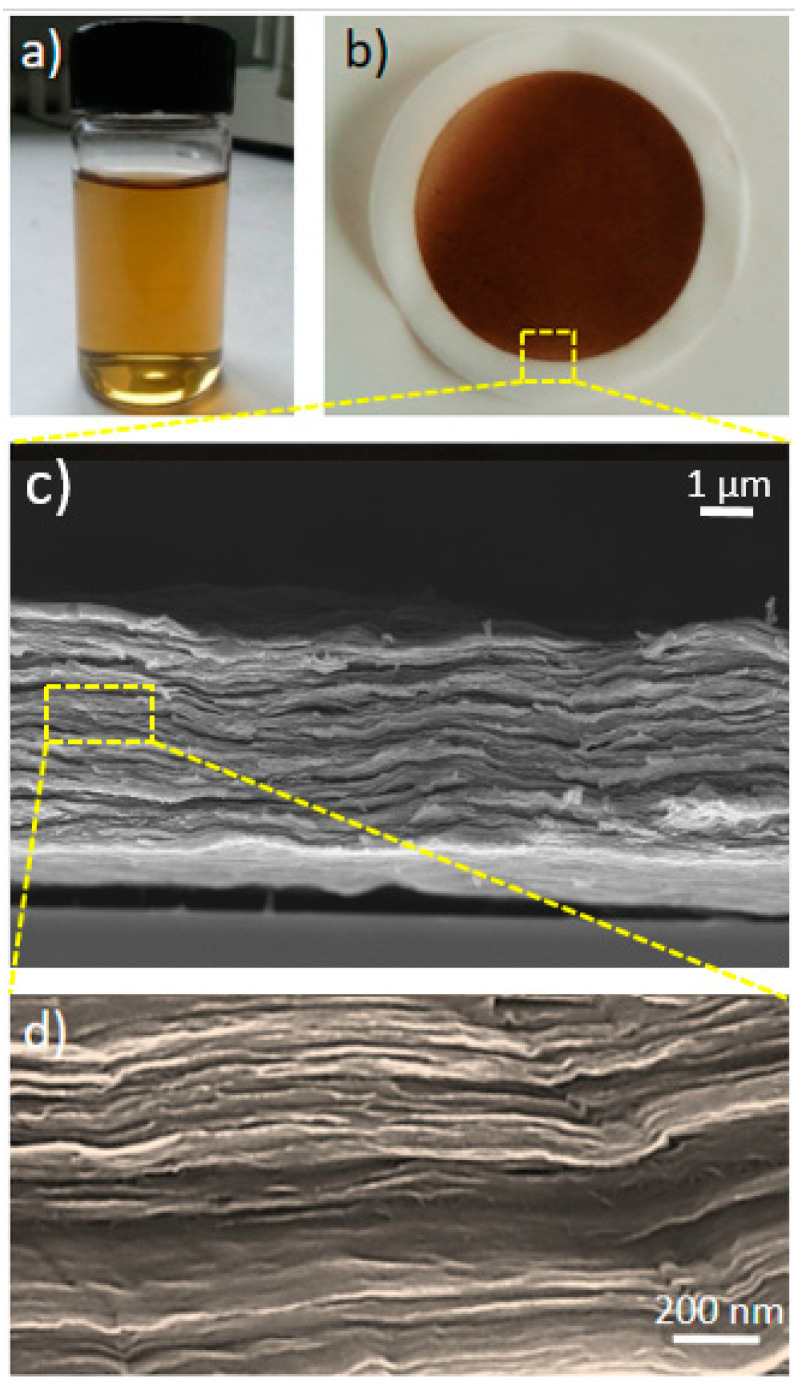
(**a**) GO dispersion in water. (**b**) GO membrane on polycarbonate track etched (PCTE) support. (**c**) FESEM image showing a cross-section of a self-standing GO membrane without polymeric support. (**d**) Higher FESEM magnification showing the layered ordered structure of such membranes.

A good control of the GO membrane’s structure is needed to achieve stable and high-performance membranes. Since the real structure of these membranes is far from the ideal case of perfectly stacked layers [43], a lot of attention must be devoted to the preparation method. The first parameter analyzed to improve the GO membrane filtration properties is the initial concentration of GO flakes in water. A concentration of 1 mg/mL was chosen for the preparation of all the membranes (Figure 2a,b). In fact, concentrations above 1 mg/mL lead to an insufficient dispersion of GO flakes, resulting in aggregates in water and a consequent poor uniformity of the final membrane, while, on the other hand, concentrations below 1 mg/mL show only a faster dispersion of GO flakes in deionized (DI) H**_2_**O without particular effects on the filtration properties. The second examined parameter is the thickness of the GO membrane; a thinner membrane gives higher flux, but can also give micro and macroscopic defects, lowering the filtration efficiency [44]. In trying to maximize the flux while reducing the defect occurrence, the best results are reached with a GO loading on the support layer equal to 0.25 mg/cm^2^, corresponding to a membrane thickness of 700~800 nm. Lower GO loadings led to diffused defects and poor filtration properties, while thicker membranes with a higher GO loading only reduced the water flux without providing higher values of rejection (see Figure S3 in the Supporting Information).

An appropriate choice of the fabrication technique is essential, because this strongly affects the final structure of the membrane [45]. In this work, we investigated both vacuum filtration and pressure-driven filtration. In the first case, the support layer is laid over a grid and the feed solution containing GO flakes is poured on the top. Below the grid, there is a chamber connected to a membrane pump generating a low vacuum (100 mbar). When the pump is turned on, the water starts crossing the porous support because of the pressure difference. When the process is completed and no water remains on the feed side, a compact layer of GO is found on top of the porous substrate, as shown in Figure 2c,d. An analogous result can be obtained with a dead-end apparatus, which allows overpressure to be applied on the feed side by pushing the water to pass across the support grid and the subsequent deposition of the GO flakes. In both cases, it is important that the supporting grid has a microporous structure, avoiding macroscopic holes larger than few hundred micrometers which generate an irregular water flux on the polymeric substrate, leading to a poor uniformity in the final GO coating. In our case, the best conditions for vacuum filtration were found using a microporous glass support (see Figure S4 in the Supporting Information). Instead, for the dead-end apparatus a microporous metal disk proved to work properly in sustaining the applied pressure and granting a good uniformity to the GO membrane. The GO coatings obtained from vacuum filtration have a diameter of 40 mm, while the ones obtained with the dead-end apparatus have a diameter of 43 mm, with the difference relying on the apparatus assembly.

The last point to be carefully considered during the formation of a GO membrane is the drying step. The transition from wet to dry is crucial for GO, since a lot of water molecules are trapped inside its hydrophilic structure (refer to Table S2 in the Supporting Information). During the drying step, the water molecules will find a path to leave the membrane as a vapor. At ambient temperature or upon heating, the vapor molecules can crack the membrane’s structure to create an escape route, which can be orders of magnitude larger than the interlayer distance between the GO layers (see Figure S5 in the Supporting Information). Such defects weaken the membrane’s structure, which can be broken by the pressure applied during the filtration tests or at least can lead to a lowering of the rejection.

To reduce the amount of water trapped inside the membrane, first of all it is necessary to use a support layer which does not absorb water. For this reason, cellulose nitrate (used for stability tests) has been discarded. Anodized alumina oxide (AAO), employed for the proof of principle, showed poor water retention but also poor mechanical properties (too fragile). Polyether ether ketone (PEEK) was the best suited for harsh environments, but required a pressure above 7 bar to force the water to pass through, lowering the efficiency of the system. Finally, hydrophilic polycarbonate track etched (PCTE) porous membranes with nominal pores of 100 nm were chosen because they did not trap a high amount of water, while contemporarily showing good mechanical properties. Moreover, a pore diameter of 100 nm was enough to grant a water flux orders of magnitude higher than the GO membranes, while avoiding GO flakes passing through. Instead, in choosing highly hydrophobic supports as in the case of polypropylene (PP), it was not possible to coat hydrophilic GO flakes as they were simply sliding on top of the polymer.

After selecting the proper substrate, the fabrication method was optimized. In the case of vacuum filtration, the membrane must be left under a vacuum inside the apparatus until most of the water is removed and the membrane appears dry to the naked eye. In this case, even though most of the cracks linked to evaporation are avoided, we experimentally verified the consequence of the situation reported by Tsou et al. [43] regarding the self-assembling of the GO flakes. The membranes produced by vacuum filtration show a rejection of toluene of only 60~70%. Such a value is 20~30% lower than that obtained with membranes produced by pressure-driven filtration. Indeed, using a dead-end apparatus improves the GO flakes’ stacking because the pressure-driven filtration grants a constant pressure on the top of the membrane for the whole process. In the vacuum filtration method, the pressure drop across the developing membrane increases in magnitude as the thickness of the GO layer increases. Similarly, with regard to the pressure-driven filtration, the pressure must be applied until all the water is removed from both the chamber and the membrane. In particular, using a dried gas source like pure nitrogen, no water vapor will be left in contact with the membrane, preserving its dry state also after the end of the process. Indeed, we experimentally observed that a fully dried GO membrane is completely impermeable to nitrogen, at least for pressures up to 4 bar. The GO membrane, after its formation, was left inside the apparatus with an applied N_2_ pressure of 4 bar until the water was completely collected in the second chamber. The N_2_ line was then closed, checking that no leakage at all could be addressed to the gas connections. After 2 weeks, the gas pressure remained constant. Similar results have been previously reported in the literature for a hollow fiber coated with a 700 nm-thick GO layer tested at ~5 bar [46] and for a 10 µm-thick self-standing GO membrane at a pressure of 0.1 bar [47]. Our finding provides a further confirmation that dry GO membranes are impermeable to N**_2_** even at high pressure values. However, once exposed again to liquid water during filtration, the GO membrane loses its impermeability to N_2_. In conclusion, to preserve the dried condition and grant the best filtration properties, we decided to work with GO membranes prepared in the dead-end apparatus on PCTE, removing the N_2_ overpressure just before pouring in the feed solution to be tested for filtration purposes.

### 3.2. Membrane Characterization: Interlayer Distance, Z-potential, Surface Area

**Figure 3:**
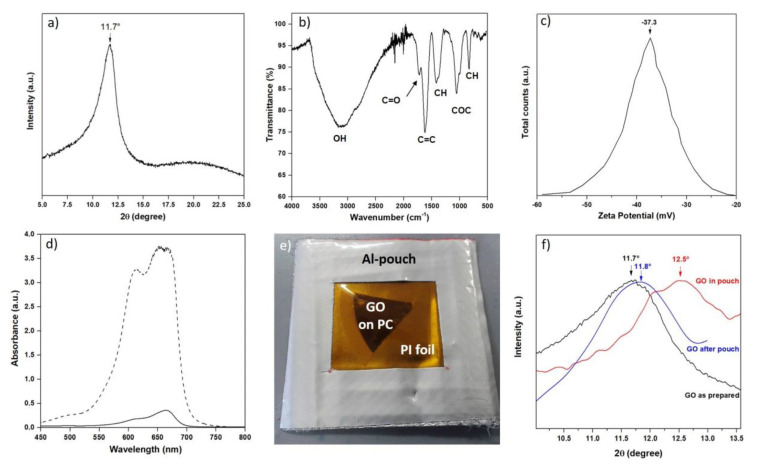
(**a**) XRD spectrum of GO membrane: the peak linked to interlayer distance is located at 11.70 degrees (d = 7.56 Å). (**b**) FTIR spectrum of GO membrane. (**c**) Z-potential measurement in GO solution. (**d**) UV-Vis spectrum of methylene blue (MB) solution before adsorption (dashed) and after adsorption (continuous) by GO membrane. (**e**) Digital image of the polyimide-modified Al-pouch containing a piece of dry GO membrane. (**f**) XRD spectra showing the evolution of the GO interlayer peak before, during, and after sealing in the pouch (see Figure S6 for further details).

From XRD analysis performed in ambient conditions, the interlayer distance (*d*) of GO is found to be 7.6 Å (Figure 3a), with a standard deviation of 0.1 Å. Such a channel height allows GO membranes to be used for NF, ideally rejecting every molecule with dimensions larger than *d*. However, the *d* value measured from XRD should be corrected, taking into account, on one side, that it includes the thickness of a GO layer (comparable to the one of pure graphene—i.e., 3.4 Å). On the other side, an increase in the *d* value has to be expected due to the relative humidity; such an increase has been reported in the literature and it has been demonstrated to reach the maximum value of 3 Å for a membrane completely immersed in water [48]. Therefore, since these two effects compensate each other, we decided to consider the *d* value measured with XRD as the average thickness of the channels during filtrations.

FTIR spectroscopy (Figure 3b) was performed with the purpose of checking the type of functional groups present in our GO membranes. FTIR analysis highlighted the presence of hydroxyl (~3100 cm^−1^), carbonyl (1730 cm^−1^), alkenyl (1628 cm^−1^), and epoxy (1061 cm^−1^) groups. Such groups result in a net surface charge of the GO dispersion with a Z-potential (Figure 3c) equal to −37 ± 1 mV, in accordance with the previous literature [49]. Such a value is responsible for the high stability of GO dispersions in water, since the Z-potential evaluates the electrostatic potential near the surface of suspended particles. Consequently, agglomeration in water is prevented by electrostatic repulsion among GO flakes, caused by the presence of negatively charged groups on the flakes’ surface.

Methylene Blue (MB) is a positively charged molecule chosen to evaluate the surface area of the GO due to its affinity to the negatively charged surface of GO. After leaving a self-standing GO membrane inside a solution of MB 100 µM for two days, UV-Visible spectroscopy was employed to evaluate the remaining fraction of MB in the solution (refer to Figure S7 for further details). Knowing the area of the molecule [50] and the mass of GO, the surface area of GO can be estimated. From the MB adsorption (Figure 3d) technique, the surface area of our GO membrane is found to be 1520 m^2^/g. This value is higher than the BET results reported in the literature, ranging from ~400 [51] to ~900 m^2^/g [52], but far from the theoretical surface area of 2630 m^2^/g computed for pure graphene [53]. Comparing the value obtained from MB absorption with other works [54,55], our GO membranes exhibit a high surface area, preserved also in this stacked configuration, suggesting the presence of a high number of channels and active sites.

A further test was performed in order to better investigate the *d* increase upon wet conditions and to explain the origin of the nitrogen impermeability. A GO membrane was prepared as previously described using the dead-end apparatus. Once all the water was passed through the polymeric support and the GO layer was formed, the dead-end tank was kept under nitrogen pressure at 4 bar. It was opened inside a dry room (relative humidity lower than 0.5%), slowly releasing the overpressure, and the membrane at the bottom was immediately removed and sealed inside a Kapton-modified laminated pouch under vacuum conditions to prevent hydration (Figure 3e). The Kapton window was added to the standard laminated Al foil to allow XRD investigation. The interlayer distance of GO was found to be 7.0 Å (Figure 3f), which corresponds to a reduction of 0.6 Å. Taking into account the thickness of a GO layer (3.4 Å), which cannot be varied, the decrease in dimensions can only be addressed to channel shrinking, going from 4.2 to 3.6 Å. After opening the pouch and keeping the membrane in ambient conditions for 2 h, the XRD measurement was repeated (Figure 3f), finding 7.5 Å as the interlayer distance and confirming the key role of the humidity in the channel dimensions. Therefore, the experimentally observed hindered nitrogen permeation must be addressed to this shrinkage, caused by the absence of water molecules usually responsible for enlarged channel dimensions.

### 3.3. Water Permeation Measurements

**Figure 4:**
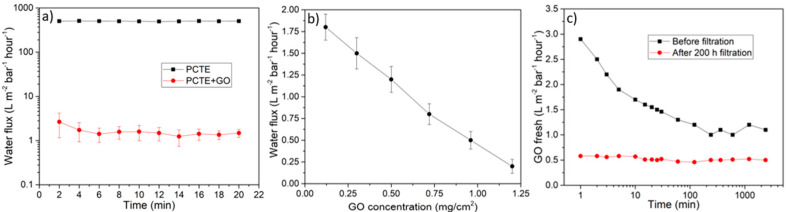
(**a**) Water flux comparison between the support layer alone (PCTE) and the support layer with the GO coating on top. Error bars are evaluated as the standard deviation of 10 different samples. Error bars on PCTE cannot be displayed in this scale. (**b**) The water flux through GO membranes with different thicknesses (reported as GO loading) at 1 bar of applied pressure is shown. (**c**) Water flux measured up to 2500 min on the same membrane, before filtration tests and after 200 h of operation.

Flux tests were performed on GO membranes employing DI H**_2_**O. The results are shown in Figure 4a, where the obtained water flux is reported as a function of time for the bare PCTE support layer and for the GO membrane on PCTE. The result shows that the GO coating (whose thickness is around 750 nm) leads to a flux reduction of at least two orders of magnitude. This value was confirmed by repeating the experiment with 10 different GO membranes prepared in the same conditions. We also tested different thicknesses of GO coatings, experimentally verifying an almost linear dependence of the flux on the membrane thickness (Figure 4b). In the literature, thinner GO membranes in the order of tens of nm are reported to have a really high flux in the order of 10^2^ L bar^−1^ h^−1^ m^−2^ [10,56]. However, recent studies by Chong et al. [43,46] have reported values comparable to our findings and provided an explanation of the mechanism behind the dramatic flux reduction during filtration experiments, which was already reported in the literature [10,44]. They investigated the effect of the water permeation and drying process on GO membranes, discovering that high flux is achieved only in the presence of highly disordered membranes—i.e., membranes whose structure is far from the ideal parallel flake stacking. They also proved that the origin of such a disorder must be addressed to the drying step performed in air. Finally, we also measured the water flux of a GO membrane before and after a filtration test that lasted for 200 h (Figure 4c). It was possible to see how, before operation, the water flux kept decreasing. As previously stated, this mechanism is associated with the stabilization of the membranes’ structure. After 200 h of operation, the water flux was perfectly stabilized at a slightly lower value with respect to the initial conditions. This result highlights how, after 200 h of operation conditions, the membranes are stable and able to keep a steady flux, a crucial requirement for an industrial plant.

### 3.4. Stability Test

The pressure applied for all our tests is 1 bar. Higher pressures up to 25 bar were applied to check the mechanical stability of the membranes. To do this, the pressure was gradually raised from 0 to 25 bar, stopping every 5 bar for 10 min. After that, the pressure was brought back to 1 bar, and the flux test was repeated. The results obtained were in line with the ones reported in Figure 4a, confirming the good mechanical stability of the GO membranes and proving that they are able to work in a wide range of pressures.

Stability tests were carried out for 3 months, leaving the GO membranes immersed in different solutions and periodically checking the membrane conditions. The results are reported in Table 2. No macroscopic damage was observed in the presence of pure water, acidic solutions, salt, and organic solvents. The only effect observed is a flux reduction up to 70% in the case of acidic solutions. Instead, a long-term immersion in a basic solution leads to diffused damage to the whole GO membrane (see Figure S8 in the Supporting Information). In particular, those membranes over time became more and more fragile. After 1 month, the membrane began to crack, and damage on the edges are clearly visible. After 3 months, it was completely destroyed, having again the GO as water dispersion and no more as a stacked membrane. However, fluxing concentrated solutions (0.5 M) of both acids and bases for 20 min showed no significant changes in the flux and rejection properties. Indeed, such treated membranes attested their water flux in the range reported in Figure 4a, while their toluene rejection was in line with the results reported in Section 3.5, meaning that the GO membranes can withstand the procedures commonly used for membrane cleaning. The GO membranes also proved to be compatible with salty water, resulting in no change in rejection properties in both cases (see Table S3 in Supporting Information).

### 3.5. Filtration Measurements

**Figure 5:**
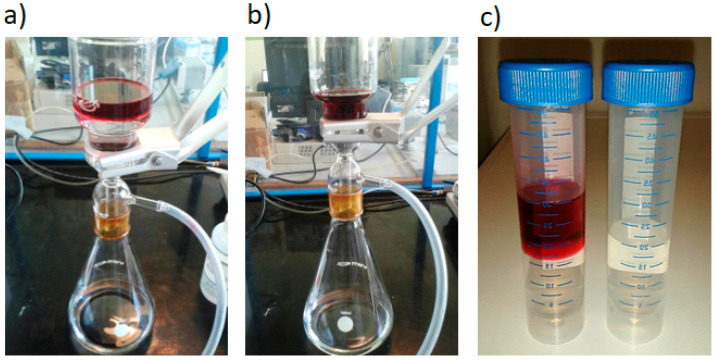
(**a**) Initial conditions of the proof of principle, where the toluene/Oil Red O film floats over DI H_2_O. (**b**) Vacuum filtration using a GO membrane on anodized alumina oxide (AAO) support; only water is collected below. (**c**) Permeates collected by vacuum filtration in (**a**,**b**) conditions. On the left, a solution of toluene and DI H_2_O filtered with a simple AAO membrane. On the right, the same solution filtered with an AAO membrane coated with GO. No toluene at all was found able to pass through the membrane in the second case.

A preliminary proof of principle for the selective filtration of toluene employing GO membranes was performed. Initially, a solution made of DI H_2_O and toluene (50% *v/v*, with toluene floating over water) was filtered by an anodized alumina oxide (AAO) membrane using a vacuum filtration setup. Both toluene and water were found able to permeate through. Then, the same procedure was repeated with an AAO membrane coated with GO, and only water was found to be able to permeate through the membrane; the toluene was completely rejected (see Figure S9 in the Supporting Information). For better evidence of toluene rejection, Oil Red O was added to the initial solution. In fact, this dye is insoluble in water and its attachment to the toluene molecules gave a typical and well visible red coloration (see Figure 5a–c).

This experimental evidence allowed us to move to the next part of the preliminary tests, in which the toluene was present below its solubility limit in water (which is equal to 526 ppm [57]). The aim was to study the GO membranes’ rejection properties towards dissolved contaminants and not only towards the concentrated phase or the stabilized emulsions which are usually investigated in the literature [31,32]. Accordingly, the dead-end apparatus, with an applied pressure of 1 bar, was employed for pressure-driven NF tests to measure the rejection percentage, defined as:

(1) $$R=\frac{\left(Feed\;Initial\;Concentration-Permeate\;concentration\right)}{Feed\;Initial\;Concentration}\cdot 100,$$

where the feed initial concentration was measured before starting the filtration. The rejection evaluated in this way will be underestimated because it does not take into account the fact that the concentration of the feed increases over time; moreover, the concentration polarization is not taken into account. Nevertheless, such a rough estimation could be enough to provide evidence for the possibility of employing GO membranes for the removal of BTX contaminants from oil and gas wastewater.

Solutions of toluene in DI water at a concentration of 100 ppm were filtered with GO membranes and the rejection was evaluated. For all the experiments, the feed side of the apparatus housed 200 mL of solution. During the experiments, only half of the solution was filtered to be consistent with an industrial situation in which a continuous flow is present and high concentrations are never reached on the feed side. For the same reason, each membrane was reused three times. The results are reported in Table 2. Each value has been obtained as the mean of at least five different GO samples to guarantee the repeatability. The results showed a rejection higher than 80% for a starting concentration of 100 ppm.

Starting from this promising result, we moved to the real goal of this study, which was measuring the rejection towards BTX molecules at a concentration of only 1 ppm, as representative for the contamination of BTX present in industrial wastewater. It is important to underline that these results were obtained with a single-step filtration procedure. In fact, higher rejection rates could be achieved by using a stack of GO membranes; starting from a feed concentration of 100 ppm, the ppb regime can be reached within three filtration steps.

To further investigate the selection mechanism, methylcyclohexane was employed as a new probe molecule for filtration tests. In fact, the MCH molecular structure is the same as that of toluene, apart from the aromatic double bonds that are, in this case, saturated. Therefore, the steric encumbrance is the same, but the MCH is expected to have lower interaction with the negative charges of GO. The pressure-driven NF experiment should, in this way, allow us to assess whether the toluene rejection is mainly linked to electrostatic repulsion or to other causes. The results show an increase in rejection of 7% with MCH molecules. It follows that the rejection of toluene must be addressed to other reasons, such as its water solubility, leading to a poor capability to pass through a channel (whose dimensions are comparable to the molecule’s diameter) filled with water molecules, similarly to the case of ion filtration reported in the literature [58]. Moreover, the higher rejection of MCH with respect to toluene allows us to exclude the possibility that toluene could be trapped inside the GO structure due to adsorption on the GO surface caused by the π–π stacking of the toluene aromatic ring on the non-defective regions of GO. In such a case, a lower rejection of MCH would have been observed, with all sp^3^ bonds of this molecule not interacting with the GO surface. In addition, a rejection approaching 100% would have been expected moving from 100 ppm to 1 ppm in the case of adsorption, with the membranes having the same thickness in all tests and, therefore, statistically the same amount of adsorption sites. A further confirmation of this hypothesis came from the GC-FID analysis of feed solutions, which reported an increase in the concentration of the probe molecules proportional to the amount rejected by the membrane.

A deeper investigation was performed, by testing different organic compounds. Common organic solvents with a dipole moment an order of magnitude higher than toluene and MCH [59] were chosen for a second set of experiments; acetone, alcohols such as methanol and ethanol, and glycols such as ethylene glycol and triethylene glycol were mixed with DI H**_2_**O in a range of 5~30% *v/v*. The reason for such high concentrations lies again in the solubility limit of these compounds; their molecules possess a high affinity to water and the goal of this set of filtration tests was to study the selectivity of GO membranes towards highly miscible compounds in water. The results show practically no rejection for all these compounds apart from TEG, for which, however, the rejection value is very low. In fact, the TEG molecules exhibit not only the highest dipole moment but also the largest dimensions (Table 3). Filtration tests of toluene and triethylene glycol were also performed in the presence of NaCl 0.6 M, with the aim to simulate seawater commonly present in offshore fields for both IOR and EOR, leading to the same rejection values towards such compounds (refer to the Supporting Information for further details). Finally, the influence of bare PCTE polymeric substrate was tested by filtering the toluene (100 ppm and 1 ppm) and MCH (1 ppm) solutions. No rejection at all was observed in any of the cases.

We addressed the observed behavior of the membrane to the natural hydrophilicity of GO. Indeed, the presence of many oxygen-containing functional groups on the edges and inside the channels of the membrane are responsible for the water affinity of GO. Therefore, they facilitate the permeation of molecules with high dipole moments alike, as described in the literature in the case of methanol purification [61,62]. Such molecules are able to move almost unimpeded inside the channel, interacting with both the functional groups of the GO membranes and the water itself, modifying their hydration shell (and therefore, their steric encumbrance) accordingly to the interaction with these groups. For the same reason, non-polar molecules are rejected since their mobility inside the channels is strongly hindered, even though their diameter is slightly lower than the average channel dimension. Therefore, most of the non-polar molecules are rejected in the very beginning part of their path through the channels, meaning that an ideal defect-free GO membrane would require only a few layers to achieve the same rejection, reducing costs while increasing the flux. These findings implicate that GO membranes are suited for a cross-flow filtration setup in which BTX contaminants and other non-polar hydrocarbons are the target molecules, even if present at a concentration of only 1 ppm in water.

## 4. Conclusions

This study provides experimental proof of the possibility of using GO membranes as NF membranes for the removal of BTX contaminants present in water below their solubility limit. We reported how to prepare reliable membranes with a simple and scalable method. The filtration results are promising, showing a rejection higher than 80% for a concentration of toluene around 100 ppm, and a rejection above 90% for lower concentrations corresponding to 1 ppm or below. We also proved that GO membranes can be efficiently used to filter other hydrocarbons, such as methylcyclohexane, while we experimentally observed the absence of selectivity towards polar molecules such as alcohols and glycols.

We also found that a completely dried GO membrane is impermeable to nitrogen, at least for pressures up to 4 bar, exhibiting a reduction of about 0.6 Å in the interlayer distance among GO flakes. Herein, we demonstrate that, by using N**_2_** pressure to filtrate the GO solution in a dead-end apparatus, a low interlayer distance can be achieved directly during the membrane fabrication step.

To conclude, the results show how GO membranes can be employed in oil and gas and other industrial applications because of their scalability, good stability, and high selectivity, allowing them to meet the requirements for industrial wastewaters of many countries. GO membranes, depending on the application, can be employed to satisfy the increasingly stringent regulations (this is the case of the Italian Legislative Decree No. 152, approving the code on the environment) or to increase the industrial production, thanks to the lower amount of hydrocarbons discharged in water (as in the case of the United States Environmental Protection Agency’s water quality criteria, under the National Pollutant Discharge Elimination System permission, regulated by the Code of Federal Regulation 40 CFR § 122, 141, 435 and 33 CFR § 151.A).

## Figures and Tables

**Table 1:** List of recent works in the separation field employing different kinds of membranes, techniques, and concentrations of oil-in-water emulsions. The aim of this table is to underline the lack of tests in the literature of filtrations below the solubility limit, avoiding the presence of emulsions and surfactants.

Membrane	Solution	Concentration	Separation	Source
Al_2_O_3_/GO	Machine oil	1 g/L	Crossflow	[31]
GO/polymers	Mineral oil, toluene, hexane, chloroform	3.33 g/L	Vacuum	[32]
PAN/GO	Lubricating oil	1 g/L	Crossflow	[33]
Polymeric	Kerosene	50 g/L	Tangential flow	[35]
TiO_2_/ceramic	Crude oil	200 mg/L	Crossflow	[37]
Al_2_O_3_/ZrO_2_	Cutting oil	5 g/L	Tangential flow	[38]
GO	H**_2_**O in EtOH	0~100%	Pervaporation	[40]
Polymeric	H**_2_**O in hexadecane	97%	Crossflow	[41]
GO	Toluene, methylcyclohexane	1 mg/L	Crossflow	This work

**Table 2:** Results of the stability tests of GO membranes in different solutions for 3 months. “None” refers to a membrane that, after the stability test, is not only compact at visual inspection, but is still possible to be used without cracking its structure during the filtration test. Refer to Table S4 in the Supporting Information for further details.

Solution	Concentration	Effect
DI H**_2_**O	100%	None
HCl	0.01 M	None
HNO_3_	0.01 M	None
NaOH	0.01 M	Damage
NaCl	0.6 M	None
Toluene	100 ppm	None
Ethylene glycol	10%	None
Ethanol	10%	None
Acetone	10%	None

**Table 3:** Comparison of the rejection properties of GO membranes towards different molecules, all of them dissolved in water in concentrations below their solubility limit [60]. In all the cases, a pressure of 1 bar is applied on the feed side. The support layer chosen for all the measurements is PCTE.

Molecule	Molecular Diameter (nm)	Dipole Moment (D)	Concentration	Rejection (%)
Toluene	0.696	0.31	100 ppm	84 ± 4
Toluene	0.696	0.31	1 ppm	90 ± 2
Methylcyclohexane	0.740	0.00	1 ppm	97 ± 1
Methanol	0.505	2.87	5~30%	<5
Ethanol	0.570	1.66	5~30%	<5
Acetone	0.615	2.69	5~30%	<5
Ethylene glycol	0.561	2.27	5~30%	<5
Triethylene glycol	0.751	2.99	5~30%	20 ± 1

## Supplementary Materials

The following are available online at https://www.mdpi.com/2079-4991/10/11/2242/s1.
