# Supplementary material for: Graphene Oxide Membranes for Trace Hydrocarbon Contaminant Removal from Aqueous Solution
Source: Nanomaterials (Basel). 2020 Nov 12;10(11):2242. doi: 10.3390/nano10112242 (PMC7697333; doi:10.3390/nano10112242)
Supplement: Supplementary file 1 [file nanomaterials-10-02242-s001.pdf]

Supporting Information

Graphene Oxide Membranes for Trace Hydrocarbon Contaminant Removal from Aqueous Solution

Alessandro Pedico [1,*], Marco Fontana [1,2], Stefano Bianco [1], Seifeddine Kara [1], Monica Periolatto [1], Stefano Carminati [3], Candido Fabrizio Pirri [1,2], Elena Tresso [1] and Andrea Lamberti [1,2]

[1] Politecnico di Torino, Dipartimento di Scienza Applicata e Tecnologia (DISAT), Corso Duca degli Abruzzi, 24, 10129 Torino, Italy; marco.fontana@polito.it (M.F.); stefano.bianco@polito.it (S.B.); seifeddine.kara@polito.it (S.K.); monica.periolatto@polito.it (M.P.); fabrizio.pirri@polito.it (C.F.P.); elena.tresso@polito.it (E.T.); andrea.lamberti@polito.it (A.L.)

[2] Istituto Italiano di Tecnologia, Center for Sustainable Future Technologies, Corso Trento, 21, 10129 Torino, Italy

[3] Eni S.p.A., Piazza Ezio Vanoni, 1, 20097 San Donato Milanese, Italy; Stefano.Carminati@eni.com

* Correspondence: alessandro.pedico@polito.it

Figure S1 shows the two filtration apparatus employed during this work.

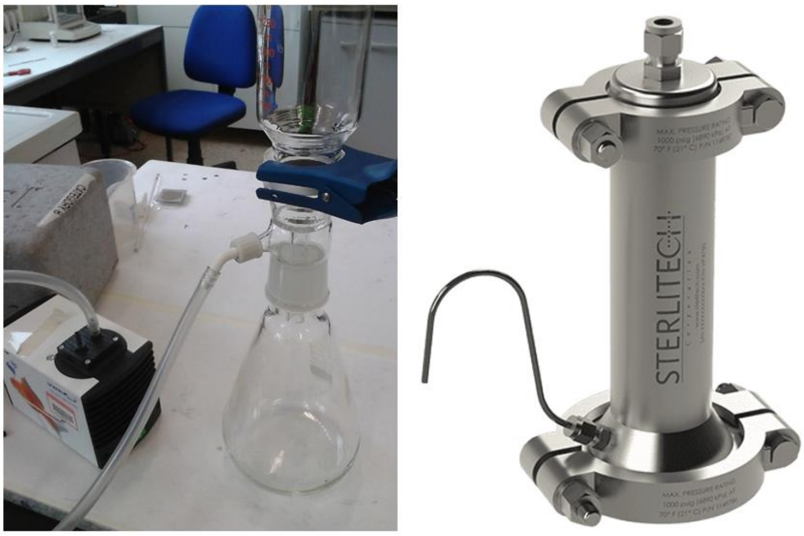

Figure S1. On the left, the vacuum filtration setup showing the rotary pump, the connecting tube, the membrane support, and the feed and permeate glassy collectors. On the right, a picture of the dead-end column.

The setup for the membrane production and filtration tests in a dead-end configuration is shown in Figure S2.

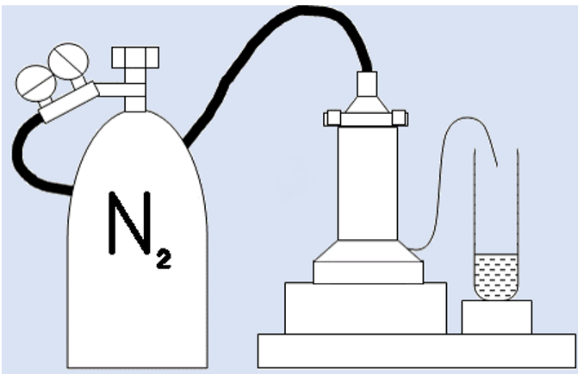

Figure S2. Schematic representation of the pressure-driven filtration process, showing the nitrogen tank, the dead-end column, and the collecting beaker.

The effect of membrane thickness over water flux through the membrane was studied to assess a possible correlation between them. The same was done for rejection. The results are reported in Figure S3, showing how the flux decreases linearly by increasing the GO thickness. The rejection did not show any improvement above a thickness of ~700 nm.

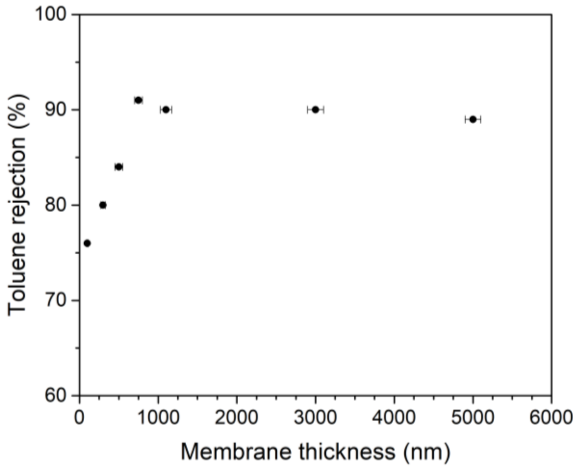

Figure S3. Effect of thickness on the toluene rejection.

In Figure S4, the three porous supports used for the membrane preparation are reported. The polymeric support layer is laid on top of them. The metallic grid, with pores of half a millimeter, is not suited for GO deposition, leading to the cracks reported in Figure S5.

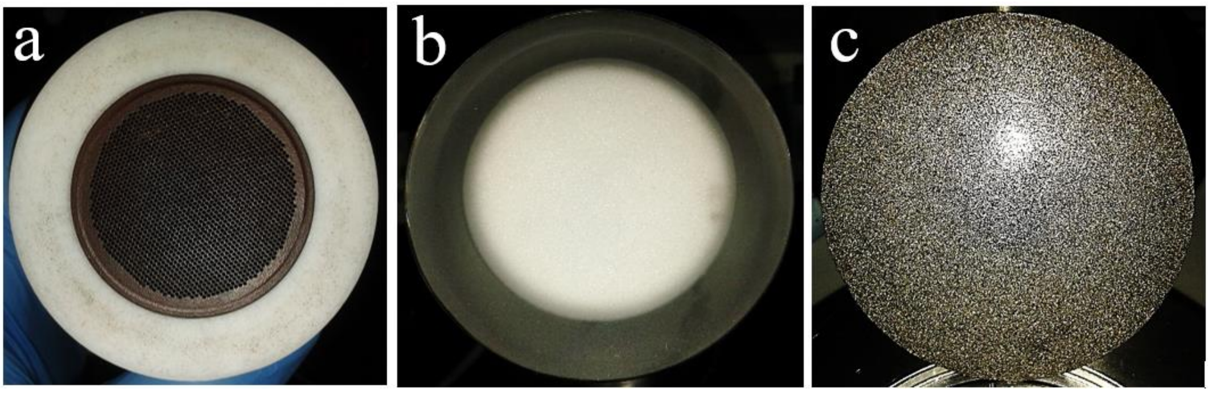

Figure S4. a) Metallic grid for vacuum filtration. **b**) Porous glass for vacuum filtration. **c**) Porous steel support inside the dead-end apparatus.

The macroscopic effect of the preparation method was studied with an optical microscope, observing the presence of defects responsible for the poor rejection (Figure S5).

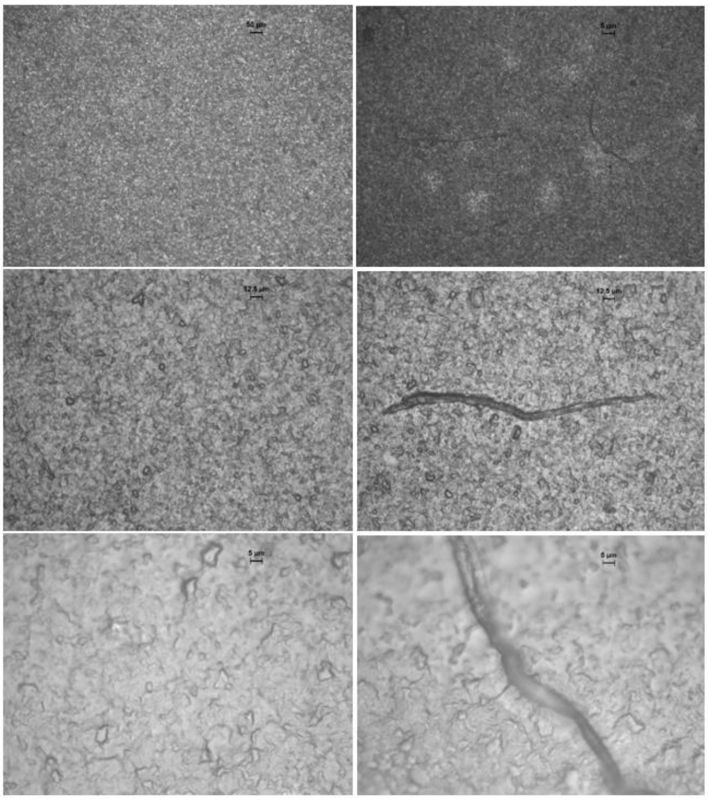

Figure S5. In the left column, the GO membrane without defects. In the right column, GO membrane with defects due to the preparation technique. The magnifications are 5×, 20×, and 50×, respectively, for the 1st, 2nd, and 3rd row.

In Figure S6, the XRD spectra of the GO membrane sealed in a vacuum inside the pouch are reported.

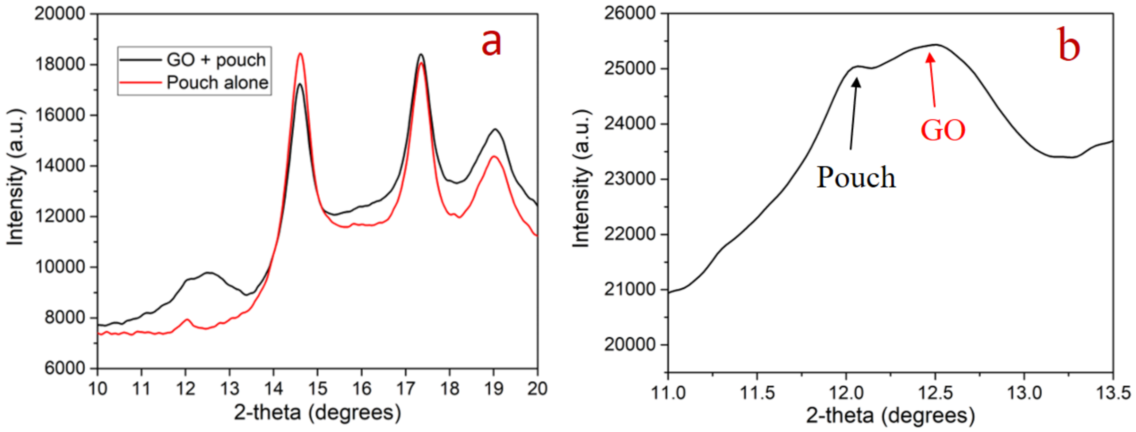

Figure S6. a) XRD spectra showing a comparison between the pouch alone and the pouch holding the GO membrane inside. A GO broad peak between 12 and 13 degrees is clearly visible. **b**) High-resolution XRD spectrum acquired in the region of the GO peak.

The calibration curve of Methylene Blue (MB) absorption using UV-Vis spectroscopy is provided in Figure S7.

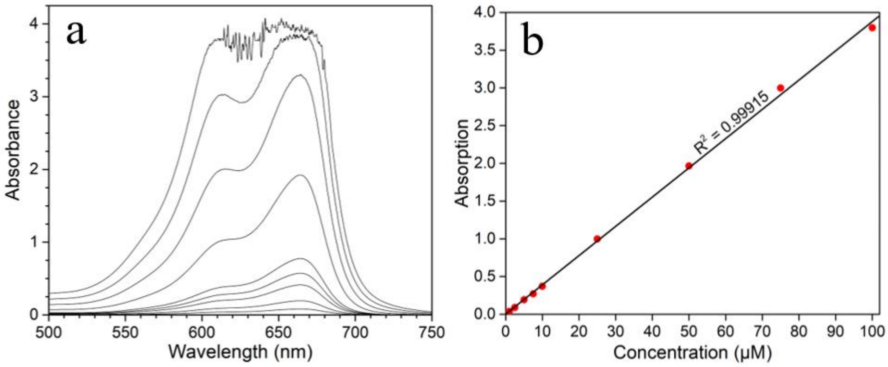

Figure S7. a) Absorption curves of MB in the visible range. **b**) Calibration curve of MB absorption at λ = 610 nm.

The stability of GO membranes in different solutions was checked every week for 3 months. While there was no visible effect for any the solvents, salts, and acids, the sample immersed in the basic solution received severe damage, which was clearly visible after just 1 month, and after 3 months it was completely destroyed (see Figure S8).

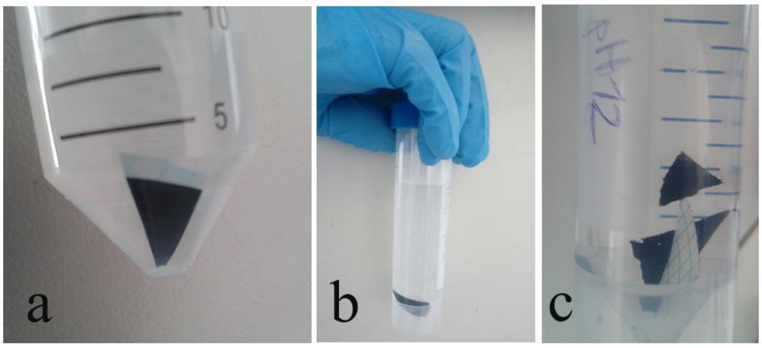

Figure S8. a) Example of GO membrane immersed in a test solution for stability purposes. **b**) GO membrane immersed in NaOH 0.01 M, day 1. **c**) GO membrane in NaOH 0.01 M, after 1 month. The membrane was detached from the polymeric support; the GO shows erosion on the edges and holes in the structure, and the initial structure broke in 3 different pieces floating inside the solution. After 3 months, the membrane was completely pulverized, showing many millimetric and submillimetric pieces floating inside the solution.

For the proof of principle, Figure S9 gives further details of what was initially observed and how it was verified experimentally.

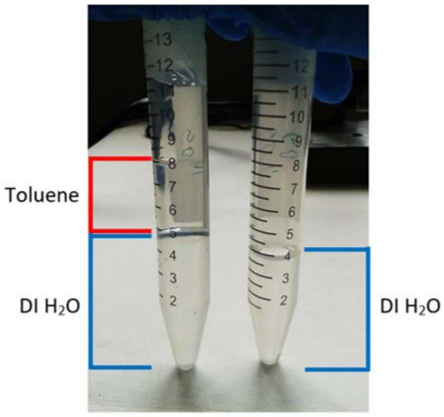

Figure S9. Proof of principle of pure toluene rejection. On the left, the permeate collected performing a filtration using only the cellulose nitrate support. On the right, the same experiment but with a GO coating on top of the cellulose nitrate. This image aims to show that the toluene rejection cannot be addressed to the dye employed (Oil Red O).

The molecular diameter of each molecule is evaluated as the double of the Van der Waals radius, defined as the mean radius of a molecule assumed as a sphere. While this is a really rough approximation, the results are quite accurate, with an overestimation of around 10% if compared to the kinetic diameter reported in the literature [1], which however refer to gases and not liquids. Even measuring the diameter of such molecules with molecular dynamics simulations using, for example, a simple program such as Avogadro [2], the values show an overestimation of 10%, which can be addressed to the fact that the Van der Waals radius takes into account also the mean distance of interaction between two molecules of the liquids. In this way, the trend is kept unchanged if compared to the above-mentioned parameters, therefore we decided to use for simplicity the Van der Waals radius, evaluated as follows:

$$r = \sqrt[3]{\frac{3c}{4\pi}\frac{MM}{d\cdot Av}},$$

where *MM* is the molar mass of the liquid expressed in g/mol, *d* is the density expressed in g/mL, *Av* is the Avogadro constant ($6.022140857 \times 10^{23}$ mol^{-1}), and *c* is the conversion factor from mL to nm^3.

Table S1. Reference values for oil and gas industries for direct discharge in water. Values taken from * Italian Legislative Decree No. 152, approving the code on the environment; ** Code of Federal Regulation 40 CFR § 141, 435.

Chemicals	Target (IT)*	Target (USA)**
Toluene	N.A.	≤1 ppm
Total Organic Aromatic Solvents	≤0.2 ppm	≤10 ppm
Total Hydrocarbons	≤5 ppm	≤29 ppm
Total Organic Carbon	≤160 ppm	N.A.

Table S2. On the left, the contact angle of a deionized water drop on a GO membrane. On the right, the results of contact angle measurements. Each value was obtained as the mean of 5 repeated measurements.

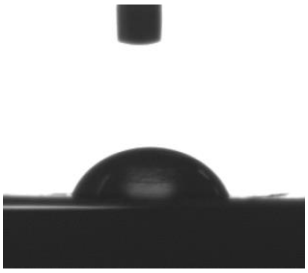

Solution	Contact Angle (°)
DI H_2O	74 ± 1
Ethylene Glycol	21 ± 2
Triethylene Glycol	20 ± 2
Toluene	9 ± 2

Table S3. Rejection in the presence of deionized water and artificial seawater. Here, a single measurement to allow a direct comparison between the two solutions is reported. In the presence of salt, the rejection is practically unchanged.

Target molecule	Concentration	Rejection (DI H_2O)	Rejection (NaCl 0.6 M)
Toluene	1 ppm	89%	91%
Ethylene Glycol	10%	0%	1%
Triethylene Glycol	10%	19%	18%

Table S4. Images showing the macroscopic effect on GO membranes reported in Table 1 in the main text.

Solution	Day 1	After 3 months
DI H_2O	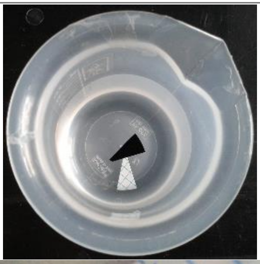	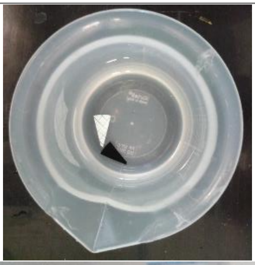
Acidic	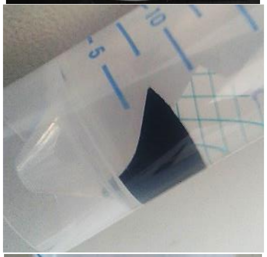	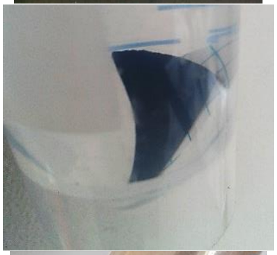
Basic	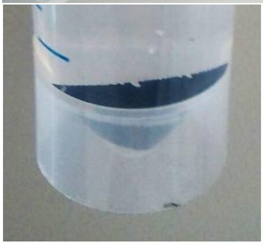	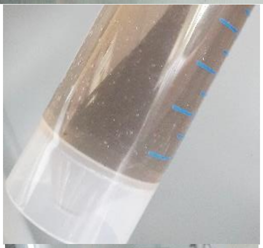
Salt	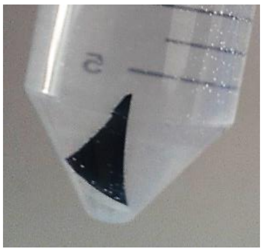	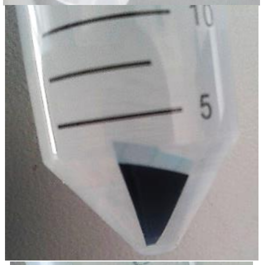
Solvents	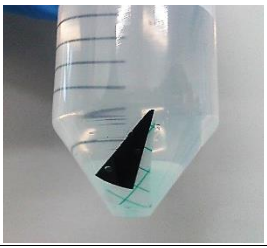	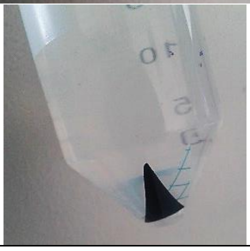

References

[1] Song, Z.; Huang, Y.; Xu, W.; Wang, L.; Bao, Y.; Li, S.; Yu, M. Continuously Adjustable, Molecular-Sieving "Gate" on 5A Zeolite for Distinguishing Small Organic Molecules by Size. *Sci. Rep.* **2015**, *5*, 13981.

[2] Hanwell, M.D.; Curtis, D.E.; Leonie, D.C.; Vandermeersch, T.; Zurek, E.; Hutchison, G.R. Avogadro: an advanced semantic chemical editor, visualization, and analysis platform. *J. Cheminformatics* **2012**, *4*, 17.
